# Etiologic Subtypes of Ischemic Stroke in SARS-CoV-2 Patients in a Cohort of New York City Hospitals

**DOI:** 10.3389/fneur.2020.01004

**Published:** 2020-09-17

**Authors:** Ambooj Tiwari, Ketevan Berekashvili, Volodomyr Vulkanov, Shashank Agarwal, Amit Khaneja, David Turkel-Parella, Jeremy Liff, Jeffrey Farkas, Thambirajah Nandakumar, Ting Zhou, Jennnifer Frontera, David E. Kahn, Sun Kim, Kelly A. Humbert, Matthew D. Sanger, Shadi Yaghi, Aaron Lord, Karthikeyan Arcot, Adam A. Dmytriw

**Affiliations:** ^1^Interventional Neuro Associates, Greenvale, NY, United States; ^2^Langone Medical Center, New York University, New York, NY, United States; ^3^Jamaica Hospital Medical Center, Richmond Hill, NY, United States; ^4^Brookdale University Hospital and Medical Center, Brooklyn, NY, United States; ^5^Neuroradiology & Neurointervention Service, Brigham and Women's Hospital and Harvard Medical School, Boston, MA, United States

**Keywords:** COVID-19, acute ischemic strokes, emergent large vessel occlusion, mechanical thrombectomy, ischemic stroke

## Abstract

**Objective:** To describe the ischemic stroke subtypes related to coronavirus disease 2019 (COVID-19) in a cohort of New York City hospitals and explore their etiopathogenesis.

**Background:** Most neurological manifestations are non-focal, but few have reported the characteristics of ischemic strokes or investigated its pathophysiology.

**Methods:** Data were collected prospectively April 1-April 15, 2020 from two centers in New York City to review possible ischemic stroke types seen in COVID-19-positive patients. Patient presentation, demographics, related vascular risk factors, associated laboratory markers, as well as imaging and outcomes were collected.

**Results:** The age of patients ranged between 27 and 82 years. Approximately 81% of patients had known vascular risk factors, the commonest being hypertension (75%) followed by diabetes (50%) coronary disease or atrial fibrillation. Eight patients presented with large vessel occlusion (LVO) with median age 55 years (27-82) and all were male. Eight patients presented with non-LVO syndromes, with median age 65.5 years (59–82) and most were female (62.5%). Both groups were 50% African Americans and 37.5% South Asian. Both groups had similar D-dimer levels although other acute phase reactants/disease severity markers (Ferritin, CRP, procalcitonin) were higher in the LVO group. The LVO group also had a significantly higher mortality compared to the non-LVO group. The most common etiology was cryptogenic (6 patients) followed by small vessel occlusion (3 patients) and undetermined-unclassified (3 patients). For the remaining 4 patients, 2 were identified as cardioembolic and 2 with large artery atherosclerosis.

**Conclusion:** COVID-19-related ischemic events can present as small vessel occlusions, branch emboli or large vessel occlusions. The most common etiology is cryptogenic. Patients with LVO syndromes tend to be younger, male and may have elevated acute inflammatory markers.

## Introduction

The novel Coronavirus outbreak came to the fore in December 2019 (1). Several features have been common manifestations of this disease: a primarily lower respiratory tract illness, a severe form that is more common in people with underlying diseases and higher mortality/case fatality in older populations (1–4). Multiple population health studies have also shown that the pandemic has impacted poorer communities with more severity than those with higher socio-economic status (5, 6). A particularly virulent form of immunological response, the cytokine storm syndrome especially seems to affect these vulnerable subgroups (4, 7, 8). This syndrome is also often associated with extrapulmonary complications of coronavirus disease 2019 (COVID-19) (7, 9).

In the last 3 months, multiple case reports have highlighted the extra-pulmonary complications of the disease (9–12). A prominent subgroup of these include thromboembolic complications (11–13). They have also been often associated with lab markers suggesting an underlying inflammatory and hypercoagulable condition (11, 13, 14). To this end, the specter of a COVID-19-specific coagulopathy has been raised, which is both inflammatory and consumptive, different from traditional disseminated intravascular coagulopathy (9).

Most neurological complications have focused on non-focal presentations like headaches, encephalopathy and skeletal muscle injury (10, 15). While some authors have mentioned the possibility of a form of meningoencephalitis, viral causation of neurological diseases have been mentioned less often (16, 17). Reports of patients presenting with focal signs secondary to ischemic strokes have been scant (10, 18). Several studies have recently shown the neurovascular effects of the virus on intracranial circulation as well as its overall prevalence in hospitalized patients (19, 20). Both have reported independently on outcomes of large vessel occlusion (LVO) as well as non-LVO patients though none have compared the two populations. Previous stroke research has shown that LVO patients tend to have worse presentations as well as outcomes than non-LVO patients (21).

The objective of this study was to study the characteristics of ischemic strokes in COVID-19 patients in two NYC hospital systems serving some of the most affected zip codes of Brooklyn and Queens (22). These centers are safety net hospitals which provide care to a high proportion of the underserved and vulnerable populations of eastern Queens as well as Central and Eastern Brooklyn. We also sought to compare the imaging, laboratory and presentation markers of different ischemic stroke subtypes seen in patients with severe acute respiratory syndrome coronavirus 2 (SARS-CoV-2) infection over a concentrated continuous 15-day period from April 1 to April 15th. We further sought to compare these variables between LVO and non LVO cases to determine if there were any factors that differentiated COVID-related strokes in these groups.

## Methods

Institutional Review Board approval as well as waiver for informed consent for the study was obtained independently from the IRBs at Brookdale Hospital University Medical Center and Jamaica Medical Center, respectively. A retrospective analysis of prospectively-maintained stroke databases was performed.

### Study Population

The period between April 1st through 15th was chosen for analysis due to universal implementation of SARS-CoV-2 screening policy for every admitted patient and peak admission rate and census for COVID-19 at these centers. Furthermore, 75% of all COVID-19 related neurovascular events for the period of March-May happened in this short 15-day window. This sample, thus, approximated with the full spectrum of disease manifestation and variability in these centers. This was also the most comprehensive and complete data we were able to collect at the time of writing this paper. All consecutive patients with ischemic stroke were chosen and results from both NYC hospitals were pooled for this analysis.

### Case Selection, Disease Definitions, and Classification

Only patients who tested positive for SARS-CoV-2 were included for this study. We excluded any cases that were thought to be COVID-19 positive based only on clinical or radiological suspicion. Tests which were administered at these organizations to confirm SARS-CoV-2 infections included: Cobas SARS-CoV-2 real time RT-PCR (Roche Holding AG, Basel, Switzerland), Xpert® Xpress SARS-CoV-2 (Cepheid Inc., Sunnyvale, CA) and Abbott Real-time SARS-CoV-2 PCR assay (Abbott Laboratories, Chicago, IL).

Acute onset of a focal neurological deficit was used as a clinical identification criterion for a neurovascular event. All patients underwent parenchymal imaging with non-contrast computed tomography (NCCT) and/or magnetic resonance imaging (MRI) of the brain to confirm evidence of an acute stroke. Vascular imaging with CT angiography (CTA) or MR angiography (MRA) was performed whenever feasible to define different types of vascular lesions. Using the aforementioned clinical definition, we identified 23 patients who sustained acute neurovascular events. We first excluded non-ischemic neurovascular events based on the initial NCCT imaging. This excluded 4 patients, two with subarachnoid hemorrhage and two with intracerebral hemorrhage, one of which was associated with venous thrombosis. Thus, there were 19 primary ischemic events detected. Based on further imaging with MRI and/or follow -up NCCT we excluded three more patients who were deemed to have transient ischemic attacks (TIA). In the period from April 1st−15th, 3,488 patients tested positive and a total of 207 patients were admitted with stroke or TIA. Of these, 27 (13.0%) were COVID positive. This yielded 16 patients eligible for final analysis comprising a pooled analysis of nine patients from Jamaica Hospital Medical Center and seven from Brookdale Hospital Medical Center.

We recorded ASPECTS (Alberta Stroke Program Early CT Score) for all patients with anterior circulation LVOs in our stroke database. Our institutional protocol is to perform mechanical thrombectomy only on patients with ASPECTS of 6 or greater. This is done in order to avoid malignant hyper-perfusion injury or for reasons of futility. However, all patients were included in the LVO category irrespective of therapy offered. Follow-up parenchymal imaging with NCCT or MRI was typically performed 2–5 days after initial presentation to define the full extent of infarcted tissue.

For the purpose of etiological classification, we utilized the SSS-TOAST classification system in order to best capture the cryptogenic etiology as well as approximate causative etiology for therapeutic reasons (23). Lesion location on CT and/or MRI was used as the criterion to define cortical vs. subcortical and/or lacunar infarctions and select small vessel occlusion etiology whenever applicable. Next, vascular imaging was used to define large artery atherosclerosis, intracranial or extracranial. Echocardiography and electrocardiogram (EKG) were used to define cardioembolic sources. When none of these were present, laboratory data was evaluated to screen for known hypercoagulable conditions. If all were negative, when patients had more than one putative mechanism or if patients had repeat infarctions despite appropriate maximal therapy (dual antiplatelet or Glycoprotein IIb/IIIa inhibitor therapy for large artery atherosclerosis and anticoagulation for cardioembolism), they were assigned the “undetermined” etiology. In all these cases, a stroke neurologist assigned the value of evident, probable or possible to the etiologic mechanism based on algorithms provided by Ay et al. (23).

### Variables Collected

Demographics including age, sex, race were collected. Past medical history, especially pertaining to vascular risk factors like diabetes, hypertension, smoking, stroke or chronic kidney disease (CKD) was also collected. COVID-19-related variables that were collected were as follows: type of symptoms, timeline from first symptoms to ischemic event, antibiotic/antiviral treatment protocol and extra-pulmonary and extra-neurological diseases. Stroke-related variables included time of onset (or last known well) and presentation time to a healthcare facility, acute parenchymal (CT or MRI) and vascular imaging (CTA or MRA), acute treatment (tPA and/or endovascular thrombectomy), pre- or post- stroke prophylaxis (anticoagulation vs. antiplatelet therapy) as well as post-stroke follow up imaging with MRI or CT. Relevant laboratory information was collected and included stroke work-up related data like routine blood counts, basic metabolic panel, coagulation profile (PT/PTT/INR), hemoglobin A1c (HbA1c), LDL, triglycerides and troponin values as well as COVID-related laboratory markers such as neutrophil-lymphocyte ratio, D-dimer, c-reactive protein (CRP), procalcitonin, ferritin, and lactate dehydrogenase. Non-neurological imaging information included chest x-ray findings, transthoracic echocardiography and deep venous thrombosis ultrasonography when available.

### Statistical Analysis

Univariable analysis of all 16 patients was performed using descriptive statistics. This included mean, standard deviation (SD), median and interquartile range (IQR) for variables with continuous distribution. Percentages were calculated for dichotomous or categorical variables. Following this, the groups were divided into LVO and non-LVO patients. Univariable analysis of intra-group variables were reported using descriptive statistics. Finally, a bivariable analysis to evaluate inter-group differences between LVO and non-LVO groups was performed. We used the Mann Whitney U-Test for continuous variables due to non-parametric distribution of both groups. Analysis was performed using the “SciPy” data science library in Python (version 3.7) and two-sided *p* < 0.05 was considered significant.

## Results

During the month of March, testing kits were limited, and universal testing had not been implemented. Also, many patients who had a terminal event within 24 h of arrival were not tested. Therefore, while 16 COVID-positive strokes were identified, the true incidence of stroke in this population remains unknown.

### Characteristics of the Study Population

The age of patients ranged between 27 and 82 years. There was an overall male preponderance 68.8% Racial distribution in descending order was as follows: African American (50%), South Asian (37.5%) and one each of Hispanic (6.3%) and Caucasian (6.3%). Approximately 81% of the patients had known vascular risk factors, the most common being hypertension (75%) followed by diabetes (50%). The majority (62.5%) of diabetics were in poor long-term control. The most common prodromal symptoms were fever and cough. Median time from first COVID symptom onset to stroke was 4 days (IQR: 7 days). The majority of our patients (75%) presented to the emergency room (ER) from the community. Four patients were admitted with respiratory symptoms and developed stroke during the hospital course. Only two patients (12.5%) presented within the window to receive intravenous thrombolytic therapy. Median time from last known well to symptom recognition was 9 h and 45 min. Median time from symptom recognition to seeking neurological care was 2 h and 16 min. Nearly a third of the patients (31%) were put on anticoagulation therapy for COVID. However, only one of them (6.3%) was on anticoagulation prophylactically secondary to elevated D-dimer levels. This patient developed a stroke in spite of full dose anticoagulation. Common laboratory derangements in our series included: elevated neutrophil-lymphocyte ratio (median, 7.2 and mean, 9.4), elevated D-dimer (median, 5,554, mean, 4,898), increased CRP (median, 14.7 mean, 24.5) and high ferritin values (median, 442.5, mean, 811.7). Involvement in other systems besides the lungs and brain was seen in 60%. Death during hospitalization occurred in 37.5%. A third of those deaths could be attributed to respiratory disease from COVID-19. No deep venous thrombosis or pulmonary embolism was detected in our series.

### Patients With Large Vessel Occlusions (LVO)

The patients with large vessel occlusion (LVO) had a median age of 55 years old (age range: 27–82 years, IQR: 23) and all were male. Vascular risk factors were present in 75% of patients, the commonest being hypertension (62.5%) and diabetes (50%) albeit with no know coronary or cerebrovascular disease. The median NIHSS for this group was 22 (IQR: 5). Only one patient was aware of being COVID-positive prior to arrival to the hospital. Three patients were admitted for pulmonary worsening and developed strokes while they were inpatients. All of them were in isolation contributing to delays in recognition of stroke. All others presented to the ER as a stroke and were found to be COVID-positive during work up of their stroke. Six patients had an echocardiogram and one (12.5%) was found to have a low ejection fraction. Three patients (37.5%) were put on anticoagulation for poststroke prophylaxis based on D-dimer levels. Another three patients were on antiplatelet agents including both patients with large artery atherosclerosis who were on dual therapy with aspirin and clopidogrel. Mortality was seen in 62.5% cases and correlated with severity of neurovascular morbidity. The mean mRS and standard deviation at discharge for this group was 5 and 1.4, respectively, while the median mRS and IQR at discharge was 6 and 2.25, respectively (Table 1).

**Table 1:** Patient with large vessel occlusion (LVO) syndromes.

**Characteristics of patients with LVO**								
Patient ID	1	2	3	4	5	6	7	8
**Demographics and vascular history**								
Age	67	69	40	46	27	55	55	73
Sex	Male	Male	Male	Male	Male	Male	Male	Male
Race	South Asian	Caucasian	African American	African American	South Asian	South Asian	African American	African American
Vascular risk factors	DM, HTN	HTN, Smoking	DM, HTN	None	None	DM, HTN, Smoking	DM	HTN, CVA
**COVID history**								
COVID symptoms	Fever, Dyspnea	Fever, Cough, Dyspnea	None	Shock, Hypoxia	Fever, Cough	Fever, Cough, Chills, Dyspnea	None	None
COVID Treatment	HCQ^*^, AZT; Doxycycline	HCQ, Doxycycline	AZT,HCQ^*^	HCQ, AZT	N/A	Ceftriaxone, AZT	HCQ, AZT	None
Other Systemic Disease	AKI, NSTEMI	AKI	N/A	N/A	N/A	N/A	N/A	AKI
**Stroke history and acute imaging**								
LKW-symptom detection (mins)	780	360	0	1,320	30	240	1,320	540
Symptoms detection -Door (mins)	0	40	905	66	97	0	200	200
NIHSS (admission)	21	21	26	32	18	23	25	6
CT ASPECTS	4	5	7	N/A	9	4	4	7
CTA/MRA lesion	Left ICA	Left M1/MCA	Left M1/MCA	Proximal Basilar	Left M1/MCA	Right ICA-T	Left ICA+MCA	Left ICA non occlusive mural thrombus without atherosclerosis f/b complete ICA-MCA occlusion
**Stroke therapy and prophylaxis**								
EVT	N	N	Y	N	Y	N	N	N
No EVT reason	Low ASPECTS	Low ASPECTS	N/A	Medically Unstable	N/A	Low ASPECTS	Low ASPECTS	Rapid infarct progression
IV tPA	N	N	N	N	Y	N	N	N
TICI	N/A	N/A	2b	N/A	2b	N/A	N/A	N/A
Stroke prophylaxis agent	Clopidogrel	UFH	ASA f/b DOAC	UFH	DAPT f/b LMWH	UFH	ASA	Eptifibatide f/b DAPT
**Imaging (non-acute)**								
CXR	B/L patchy infiltrates	interstitial opacities bilateral	Left lower field hazy opacity	B/L atelectasis	B/L patchy infiltrates	B/L patchy opacities	B/L ground glass opacities	B/L upper ground glass opacities
Ejection fraction	N/A	60%	26%	60%	75%	60%	60%	N/A
Other echo abnormalities	N/A	Mild Left Atrial dilatation	global LV hypokinesis	N/A	N/A	N/A	N/A	None
MRI brain (Follow-up infarcts)	N/A	Left MCA partial (deep + parietal)	Left Basal Ganglia, temporal gyri, frontal	N/A	N/A	N/A	N/A	Complete infarction of Left MCA territory
CT head (Follow-up)	B/L MCA territories' infarction	Left MCA deep	N/A	Right pontine/left cerebellar stroke	N/A	Right hemispheric infarction and Occipital ICH	Left hemispheric infarction	Mild early changes in left parieto-occipital areas
**Outcome and etiology**								
Etiology	Cardio: Prob	Undetermined: Crypto	Cardio: Poss	Undetermined: Crypto	Undetermined: Crypto	Undetermined: Crypto	Undetermined: Crypto	Undetermined: Unclassified^*^
mRS at discharge	6	4	3	6	3	6	6	6

*LVO, Large Vessel Occlusion; DM, diabetes mellitus; HTN, hypertension; CVA, cerebrovascular accident; Y, Yes; N, No; HCQ, hydroxychloroquine; AZT, azathioprine; AKI, Acute Kidney Injury; NSTEMI, Non ST Elevation Myocardial Infarction; N/A, Not Applicable/Available; LKW, last known well; mins, minutes; NIHSS, National Institutes of Health Stroke Score; CT, computed tomography; ASPECTS, Alberta Stroke Program Early CT Score; CTA, CT Angiography; MRA, Magnetic Resonance Angiography; ICA, internal carotid artery; MCA, middle cerebral artery; M1, M1 segment of Middle Cerebral Artery; ICA-T, ICA Terminus; f/b, followed by; EVT, endovascular therapy; tPA, tissue plasminogen activator; TICI, thrombolysis in cerebral infarction; AP, antiplatelet; AC, anticoagulation; UFH, unfractionated heparin; ASA, acetylsalicylic acid; DOAC, Direct Oral Anticoagulants; DAPT, Dual Anti-platelet Agents; LMWH, low molecular weight heparin; NLR, neutrophil-lymphocyte ratio; WBC, white blood cell count; INR, international normalized ratio; PTT, partial thromboplastin time; CRP, c-reactive protein; LDH, lactate dehydrogenase; CPK, creatine phosphokinase; LDL, low-density lipoprotein; CXR, chest x-ray; B/L, Bilateral; LV, Left Ventricle; ICH, intracranial hemorrhage; Prob, Probable; Poss, Possible; Crypto, Cryptogenic; mRS, modified Rankin scale*.

* *Patient considered to have mural thrombus of unknown etiology (no dissection flaps or underlying atherosclerotic plaque visualized on angiographic modality) with possible progression to further thrombosis and/or embolization of the entire vascular tree*.

**Figure 1:**
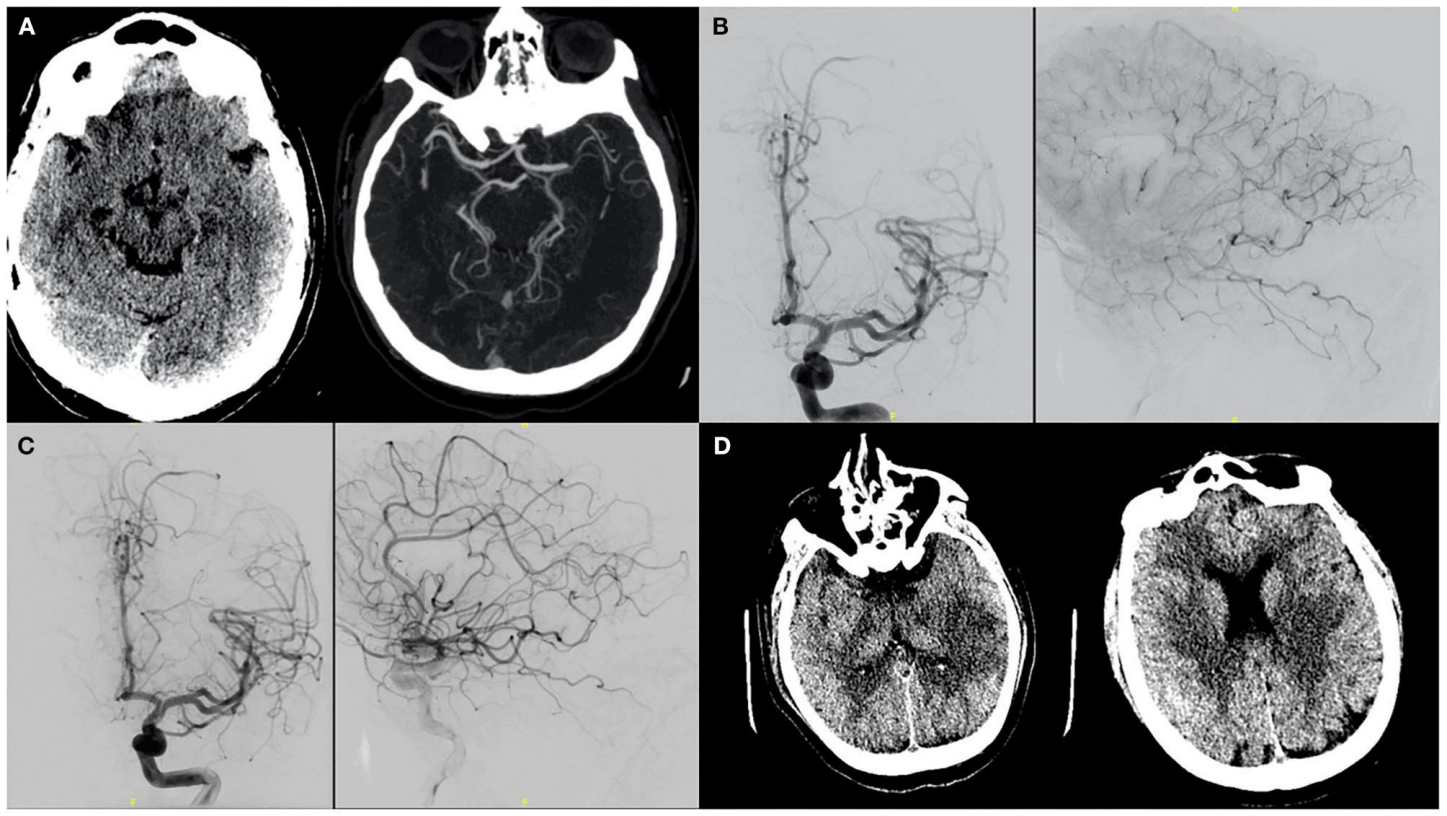
A patient with no significant past medical history presented to the ER after 8 days of viral illness and was on Azithromycin therapy after testing positive on day 3. NIHSS was 18 on presentation secondary to a left MCA syndrome. **(A)** CT/CTA confirmed a left M1 thrombus. As presentation was within 4.5 h of symptoms onset, IV tPA was administered. **(B)** The patient was taken for mechanical thrombectomy and on initial cerebral angiography showed partial recanalization post-TPA and migration of the clot to distal MCA branches. **(C)** The patient was thus given intra-arterial tPA in these branches. **(D)** Post procedurally, infarcts in the left putamen as well as temporal and parietal regions were seen. On last follow-up patient's mRS was 3.

Only two patients were able to undergo mechanical thrombectomy. One of these patients (Patient #5) both received IV tPA and underwent mechanical thrombectomy. The details of the case are discussed in Figure 1. Both patients who underwent thrombectomy were young (<45 years of age) and were initially placed on antiplatelet therapy. However, based on sustained D-dimer elevation they were later transitioned to anticoagulation. One of these patients had an EF <30%. Both were discharged to acute rehabilitation units and were able to ambulate with help at the time of discharge (mRS, 3). Of the other six, four had low ASPECTS (<6) making them poor candidates for thrombectomy. Median ASPECTS was 5 reflecting the advanced state of tissue infarction the majority of patients experienced by the time of stroke evaluation. Finally, two patients were too medically unstable for mechanical thrombectomy. One of them (Patient # 8) was initially admitted for mild stroke symptoms secondary to a partially-occlusive mural thrombus extending from the carotid bifurcation to distal cervical segment. There was no underlying atherosclerotic plaque visualized. The patient was put on IV Integrilin, however 2 h later he developed sudden pulmonary worsening leading to endotracheal intubation. Subsequent emergent neurological imaging (obtained within 3 h of initial imaging) revealed complete occlusion of the internal carotid artery (ICA) and middle cerebral artery (MCA) as well as infarction of an entire hemispheric territory.

### Non-large Vessel Occlusions (Non-LVO) Patients

Eight patients presented with non-LVO syndromes. The median age was 65.5 (range: 59–82, IQR: 14) and most of the patients were female (62.5%). Median NIHSS was 9.5 (IQR: 5.75). All had multiple vascular risk factors except one albeit with no known coronary disease or atrial fibrillation. This latter patient (Patient #8) was however morbidly obese. Only one patient (12.5%) presented early enough for IV thrombolysis. Based on parenchymal and vascular imaging, four patients were classified as small vessel disease, two with large artery atherosclerosis and two were classified as cardioembolic or cryptogenic. The latter were treated with anticoagulation while the others were treated with antiplatelet therapy. Only one patient had an EF < 30% but the presentation as well as imaging was most consistent with small vessel occlusion. The median mRS at discharge was 3.5 (IQR: 1.25) (Table 2).

**Table 2:** Patients with non large vessel occlusion (LVO) syndromes.

**Characteristics of patients with non-LVO syndromes**								
Patient ID	9	10	11	12	13	14	15	16
**Demographics and vascular history**								
Age	82	59	80	74	60	62	64	67
Sex	Male	Female	Female	Female	Female	Male	Female	Male
Race	African American	Hispanic	African American	African American	African American	South Asian	South Asian	South Asian
Vascular risk factors (description)	DM, HTN, CVA, CKD	None^*^	DM, HTN	DM, HTN	DM, HTN, CVA, CKD	HTN, CAD, CHF	HTN	HTN
**COVID history**								
COVID symptoms	Cough	Fever, Cough, Chills	Hypoxia	Cough, Chills	Cough, Chills	Fever, Cough, Dyspnea	Fever, Cough, Chills	Fever, Cough
COVID treatment	Ceftriaxone, AZT	Ceftriaxone, AZT, Remdesivir	HCQ, Ceftriaxone	HCQ, AZT	None	Ribavirin	HCQ, AZT	Ceftriaxone, AZT
Other systemic disease	AKI	Septic shock	AKI			NSTEMI		
**Stroke history and acute imaging**								
LKW—symptom detection (mins)	1,500	1,635	630	2,325	630	120	0	480
Symptom detection -Door (mins)	60	0	240	2,340	213	480	175	50
NIHSS (admission)	7	10	23	9	6	14	5	12
CTA/MRA lesion Location	N/A	N/A	N/A	N/A	N/A	N/A	Right M1-M2/MCA stenosis	Cervical LICA Occlusion
**Stroke therapy and prophylaxis**								
IV tPA	N	N	N	N	N	N	Y	N
Stroke Prophylaxis type	AC	AC	AP	AP	AP	AP	AP	AP
Stroke Prophylaxis medication	UFH	LMWH	ASA	ASA	ASA	DAPT	DAPT	Eptifibatide f/b DAPT
**Imaging and other diagnostic work Up (non-acute)**								
CXR	Right Lower Lobe infiltrates	B/L diffuse patchy opacities	B/L diffuse infiltrates	Right Lung infiltrates	Clear	Clear	B/L diffuse opacities	B/L patchy infiltrates
Ejection fraction	60%	N/A	N/A	N/A	N/A	10–15%	N/A	N/A
Other echo abnormalities	Moderate LV Wall thickening	N/A	N/A	N/A	Hyper-dynamic LV	Global LV hypokinesis/ dilatation	N/A	N/A
MRI brain (Follow-up infarcts)	Embolic post left frontal cortex	Multiple vascular territory embolic infarcts	N/A	Thalamo-capsular	Negative	Left Putamen, small right subcortical	N/A	Left Parieto-occipital watershed
CT head (Follow-up infarcts)	N/A	N/A	Internal capsule and Ganglionic infarction	N/A	Right internal capsule infarction	N/A	Right hemispheric infarction	N/A
**Outcome and etiology**								
Etiology	Undetermined: Crypto	Undetermined: Unclassified^**^	SVO: Evident	SVO: Evident	SVO: Evident	Undetermined: Unclassified^***^	LAA: Prob	LAA: Prob
mRS at discharge	3	6	4	3	2	3	5	4

*n, number of; DM, diabetes mellitus; HTN, hypertension; CKD, Chronic Kidney Disease (Stages 4-5); CVA, cerebrovascular accident; CAD, Coronary Artery Disease; CHF, Congestive Heart Failure; d, days; Y, Yes; N, No; HCQ, hydroxychloroquine; AZT, azathioprine; AKI, Acute Kidney Injury; NSTEMI, Non ST Elevation Myocardial Infarction; N/A, Not Applicable/Available; LKW, last known well; mins, minutes; NIHSS, National Institutes of Health Stroke Score; CT, computed tomography; ASPECTS, Alberta Stroke Program Early CT Score; CTA, CT Angiography; MRA, Magnetic Resonance Angiography; M1, M1 segment of Middle Cerebral Artery; M2, M2 segment of Middle Cerebral Artery; MCA, middle cerebral artery; ICA, internal carotid artery; tPA, tissue plasminogen activator; AP, antiplatelet; AC, anticoagulation; UFH, unfractionated heparin; LMWH, low molecular weight heparin; ASA, acetylsalicylic acid; DAPT, Dual Anti-platelet Agents; f/b, followed by; NLR, neutrophil-lymphocyte ratio; WBC, white blood cell count; INR, international normalized ratio; PTT, partial thromboplastin time; CRP, c-reactive protein; LDH, lactate dehydrogenase; CPK, creatine phosphokinase; LDL, low-density lipoprotein; CXR, chest x-ray; B/L, Bilateral; LV, Left Ventricle; Crypto, Cryptogenic; SVO, Small Vessel Occlusion; LAA, Large Artery Atherosclerosis; Prob, Probable; mRS, modified Rankin scale*.

** *Patient had multi-territorial infarcts in spite of full dose anticoagulation as well as elevated D-dimer (admission). Also, no echocardiography available. Therefore, unclassified due to incomplete work up as well as possible multiple etiologic mechanisms present*.

*** *Infarct patterns suggested SVO etiology but given presence of bilateral territory involvement and low EF, cardioembolism couldn't be ruled out completely. Since there were two putative mechanisms involved, based on the algorithm it was defined as unclassified*.

* *Patient was obese but without documented morbidity*.

Patient #10 was the youngest patient in this second group. This patient had no known vascular risk factors except for morbid obesity. Reason for admission was pulmonary worsening and was being treated with ceftriaxone, azithromycin and remdesivir. This was the only case started preemptively on anticoagulation with low molecular weight heparin (LMWH) based on D-Dimer elevation, with agent being enoxaparin in all cases. Due to respiratory distress, the patient has to be intubated and 27 h post-intubation developed a seizure. This prompted further imaging with MRI which revealed multiple small embolic infarcts in all three main territories with normal-appearing vasculature on MRA. This was the only inpatient stroke in this group as well as its only mortality. The latter was secondary to septic shock.

### Comparison of LVO and Non-LVO Patients

When comparing both groups, we found that both had similar racial makeup with 50% African American and 37.5% South Asian. The LVO group identified their stroke symptoms earlier and sought emergency medical care earlier than their non-LVO counterparts. Only one patient in each group presented early enough to receive thrombolysis. More patients in the LVO group were started on anticoagulation. Both groups had similar D-dimers although other acute phase reactants/disease severity markers (Ferritin, CRP, Procalcitonin) were higher in the LVO group. Counterintuitively, the neutrophil-lymphocyte ratio (NLR) was higher in the non-LVO group. Both groups also had similar levels of pre-admission diabetic control (HbA1c) as well as median number of vascular risk factors per patient. The results of analysis between both groups are summarized in Table 3.

**Table 3:** Comparison between LVO vs. non-LVO (Descriptive).

**LVO vs. non-LVO groups (Descriptive analysis)**										
	**Patients with LVO syndromes**					**Patients with non-LVO syndromes**				
**Statistical measure**	**Percentage**	**Mean**	**SD**	**Median**	**IQR**	**Percentage**	**Mean**	**SD**	**Median**	**IQR**
**Demographics and vascular history**										
Age		54	15.79	55	23		68.5	9.04	65.5	14
Sex	100.00%					37.50%				
Race	50.00%					50.00%				
Vascular risk factors (n)		1.5	1.07	2	1.25		2.13	1.46	2	2.25
**COVID history**										
Symptom duration (d)		5.75		1.5	9.5		3.33	2		
COVID awareness (Yes)	37.50%					12.50%				
Other systemic involved (Yes)	37.50%					50.00%				
**Stroke history and acute imaging**										
Location at time of CVA (Inpatient)	37.50%					12.50%				
LKW-symptom detection (mins)		573.75	526.36	450	727.5		915	816.76	630	1,143.75
Symptoms detection-door (mins)		188.5	300.03	81.5	170		444.75	780.41	194	242.5
NIHSS (admission)		21.5	7.54	22	5		10.75	5.8	9.5	5.75
Door-CT (mins)		45.67	52.49	26	42		18.67	10.78	16	11.75
**Stroke therapy and prophylaxis**										
IV tPA	12.50%					87.50%				
Stroke prophylaxis with anticoagulation	37.50%					25.00%				
Pre vs. post anticoagulation prophylaxis (Post)	100.00%					87.50%				
**Laboratory results**										
NLR		7.61	5.67	6.56	6.59		9.36	7.22	6.43	6.88
WBC (×1,000/μL)		11.1	11.2	7.5	2.25		9.03	3.45	7.85	6.1
Platelets		263.75	69.32	251	93.5		270.25	110.16	215.5	128.5
D-dimer (ng/dL)		5,323.17	4,099.19	5,727.00	6,780.50		4,473.00	3,760.67	4,134.50	6,423.50
INR		1.26	0.23	1.21	0.1		1.15	0.11	1.15	0.11
PTT (s)		29.83	4.81	29.75	1.9		36.65	16.47	30.3	3.35
Procalcitonin (ng/mL)		4.47	8.49	0.32	4.61		0.43	0.66	0.13	0.44
CRP (mg/dL)		34.78	52.42	15.15	12.33		14.33	9.96	14.7	14.65
Ferritin (ng/mL)		1,181.88	1,116.01	1,033.50	792.25		318.17	110.86	312	121
Creatinine (mg/dL)		2.57	3.08	1.5	0.97		1.29	1.01	1.05	0.78
LDH (U/L)		1,226.60	1,200.15	857	209		1,482.33	896.76	1,109.50	817
Troponin (ng/mL)		0.07	0.05	0.05	0.07		0.22	0.42	0.03	0.04
CPK (U/L)		6,909.00	16,213.29	202.5	513		616.2	591.5	267	849
LDL (mg/dL)		107.67	30.74	112	47.5		90.75	50.08	88	78.75
Triglycerides (ng/mL)		192.14	116.33	169	47.5		153	29.53	160	33
Hemoglobin A1C (%)		8.1	3.27	7.2	3.05		8.24	3	6.6	2.4
**Imaging (non-acute)**										
Ejection fraction (<30%)	12.50%					12.50%				
Etiology (Cryptogenic)	62.50%					12.50%				
mRS at discharge		5	1.41	6	2.25		3.75	1.28	3.5	1.25

*LVO, Large Vessel Occlusion; SD, Standard Deviation; IQR, Interquartile Range; n, number of; d, days; CVA, cerebrovascular accident; LKW, last known well; mins, minutes; NIHSS, National Institutes of Health Stroke Score; CT, computed tomography; ASPECTS, Alberta Stroke Program Early CT Score; CTA, tPA, tissue plasminogen activator; NLR, neutrophil-lymphocyte ratio; WBC, white blood cell count; INR, international normalized ratio; PTT, partial thromboplastin time; CRP, c-reactive protein; LDH, lactate dehydrogenase; CPK, creatine phosphokinase; LDL, low-density lipoprotein; mRS, modified Rankin scale*.

On further interrogation with bivariate analysis, we found both groups were significantly different in terms of age, gender and severity of presentation. Patients with LVO were younger, belonged to the male gender and had a higher NIHSS at presentation. The LVO group also had a significantly higher mortality. While most of the laboratory markers were not significantly different, ferritin value was significantly higher in the LVO group as compared to the non-LVO group. There was insufficient power with this sample size for determination beyond the aforementioned variables. These results are summarized in Table 4.

**Table 4:** Comparison between LVO vs. non-LVO (Mann Whitney U-test).

**LVO vs. non-LVO groups (Mann Whitney U Test)**			
**Variable**	***p*****-value**	**Median (LVO)**	**Median (non-LVO)**
Age	0.04	55	65.5
NLR	0.32	6.5625	6.4278845
WBC	0.44	7.5	7.85
INR	0.08	1.21	1.15
PTT	0.17	29.75	30.3
Procalcitonin	0.44	0.32	0.13
CRP	0.41	15.15	14.7
Ferritin	0.02	1033.5	312
Creatinine	0.13	1.5	1.05
LDL	0.23	112	88
Triglycerides	0.46	169	160
LKW-symptom detection (mins)	0.20	450	630
Symptoms detection-door (mins)	0.19	81.5	194
NIHSS (admission)	0.01	22	9.5

*NLR, neutrophil-lymphocyte ratio; WBC, white blood cell count; INR, international normalized ratio; PTT, partial thromboplastin time; CRP, c-reactive protein; LDL, low-density lipoprotein; LKW, last known well; NIHSS, National Institutes of Health Stroke Score*.

### Etiologic Classification

We used the SSS-TOAST criteria to define the etiologies of stroke encountered. We used parenchymal, vascular as well as cardiac monitoring and imaging to arrive at these diagnoses. The most common etiology was undetermined-cryptogenic (6 patients) followed by small vessel occlusion (3 patients) and undetermined-unclassified category (3 patients). For the remaining 4 patients, two were identified as cardioembolic and two as large artery atherosclerosis.

The undetermined-unclassified category included three patients. One patient (Patient #14) had infarcts in two separate vascular territories, both consistent with small-vessel occlusion as well as a putative cardioembolic source. Another patient (Patient #8) was brought in symptoms of right sided weakness with unknown time of onset and he was last known normal about 9 h ago. His symptoms were recognized in the morning, but he was brought in 3 h later to the Emergency Department of the receiving hospital. On arrival, his imaging revealed no evidence of intracranial occlusion but a partial occlusion of the left cervical ICA secondary to a mural thrombus. Three hours later while the patient was admitted to the ICU, he deteriorated secondary to his respiratory status and required intubation. We proceeded to perform an emergent MRI, post intubation, that revealed the mural thrombosis had deteriorated into complete ICA-MCA occlusion and complete infarction of the left anterior hemisphere. The third patient to be categorized as such had multi-territorial infarction in spite of anticoagulation, pre-stroke elevation of inflammatory and coagulation pathway markers as well as evidence of septic shock.

In nearly half (n, 8) of our cases, no echocardiogram was available making it difficult to objectively exclude cardioembolic etiology. In three of these cases we used parenchymal distribution to determine etiology (small vessel occlusions). Two were defined as large artery atherosclerosis based on vascular findings while one was cardioembolic due to presence of bilateral anterior circulation LVOs. Two were assigned to the undetermined-unclassified category based on reasons mentioned above (Patients #8 and #14).

## Discussion

COVID-19 patients may have an asymptomatic, moderate or a severe course of illness (12). The severe form usually requires intensive care and tends to affect patients who are older, have underlying conditions and are those who develop acute respiratory distress syndrome (24). The most severe form can also affect the heart, liver, kidneys and be associated with sepsis or septic shock (9, 12).

Several case reports have also suggested the possibility of a hypercoagulable condition caused by SARS-CoV-2 (11, 13, 25, 26). This is specifically seen in cases with higher incidence of venous thromboembolism (VTE) or pulmonary embolism (PE). Although initially thought to be secondary to poor deep venous thrombosis (DVT) prophylaxis, recent reports suggest systemic hypercoagulability at play (25, 26). This is further supported by reports of autopsies which found clots in the lung, liver as well as kidneys (12, 27). Lab markers often associated with inflammatory conditions and consumptive coagulopathies are often elevated in these cases and include elevation of D-dimer, fibrin/fibrinogen degradation products (FDP) and fibrinogen (11, 13, 14). D-dimers thresholds have indeed been used by some groups to guide prophylactic systemic anticoagulation in COVID-19 cases (28).

Most neurological manifestations of COVID-19 have been non-focal presentations such as headaches, encephalopathy, long tract cortical signs or seizures (10, 15, 16). Li et al. described the possibility of the neuro-invasiveness of SARS-CoV-2 being similar to other coronaviridae. They distinctly point out the possibility of a synaptic, but non-vascular, transmission of the virus to the brain (16, 29). However more severe forms of COVID-19 cases have been shown to present with strokes (15). As per Mao et al. the rate of neurovascular events in their series was about 5.7% of which 4.9% were ischemic strokes (10). However, direct endothelial damage mediated by the emerging role of the angiotensin converting enzyme 2 (ACE2) receptor has neither precedent in previous coronavirus epidemics nor in sepsis-induced stroke. In addition to vascular endothelial damage, acute cardiac injury and development of antiphospholipid antibodies are important contributing factors (9). In several non-US COVID-19 series, infarcts typically followed a subcortical or distal cortical distribution (10, 15). These reports also pointed at the possibility of several mechanisms for stroke that may be directly related to the infection or its complications (10, 30). These include either the causation of acute cardiac injury, creation of antiphospholipid antibodies or even the result of a severe hypercoagulable condition caused due to D-dimer or fibrinogen abnormalities.

Recently data from several institutions in the US have been published on COVID-strokes (19, 20, 31, 32). Oxley et al. reported an overall higher incidence of large vessel occlusions in COVID-19 patients who were younger than 50-years of age (19). Vascular risk factors appeared to be present in 60% of their patients and cardioembolic sources were not always ruled out. Yaghi et al. in their contemporaneous work in New York delineated a higher incidence of cryptogenic stroke, especially in those who met the criteria of embolic source of undetermined origin (20). They found this to be higher than in patients who did not have COVID-19 in both historic as well as concurrent control populations. However, the overall incidence was lower than the numbers seen in Mao et al. study from Wuhan (10, 20). They also corroborated that COVID-19 patients were younger, had higher D-dimer levels, a higher NIHSS, as well as a higher rate of large vessel occlusion. Work by Beyrouti et al. further discussed the laboratory findings and radiological features of six patients with COVID-19 (32). All of their patients presented with large vessel occlusions. They all also exhibited higher than normal values for lactate dehydrogenase (LDH), D-dimer, fibrinogen and CRP. Positivity for Lupus anticoagulant was seen in 80% of their patients though only one had a concomitant PTT elevation, something typically found associated COVID-19 hypercoagulability and antiphospholipid syndrome (33, 34).

In our series, we differentiated our population into LVO and non-LVO groups. This was done as the former is historically known to have a higher NIHSS and worse outcome neurologically when not treated (21). Our youngest patient was 27 while our oldest was 82. While the non-LVO group was older, the median ages of both groups are lower than would be expected for the general population (20). In the case of LVO, a median age of 55 is expressly unusual and was also seen in series published by other authors (19, 32). Case were 69% male and the LVO group was uniformly male which is consistent with the high male preponderance seen in COVID cases (35). Also, we had a higher representation of African American and non-white patients in our study than other investigations to-date. This may have to do with the demographic distribution of our participating institutions and further investigation is needed. These data are however instructive of how presentation may be similar in two distinct non-white populations- South-Asian and African-American.

Nearly 70% of our patients had pre-existing conditions, similar to other studies (19, 20, 32). However a higher percentage in the LVO group had no baseline risk factors, and there were no instances of known coronary disease or atrial fibrillation. We also noted a more variable time from COVID-related viral symptom onset to stroke presentation when compared to other studies with the range extending from 0 to 21 days, although the median time to presentation was 4 days. We had a higher percentage of inpatient strokes in our LVO population than our non-LVO population. Interestingly, our LVO populations were both identified earlier and sought care earlier than non-LVO populations as shown by the last known well symptom detection and symptom-detection-door time. Both of these time metrics could have been influenced by the presence of more inpatients in the LVO group. However, the arrival time for early intervention was similar in both populations as shown by rates of thrombolysis. Like Beyrouti et al. and Wang et al., we had a high rate of inflammatory markers in our stroke population (31, 32). These included NLR, ferritin, D-dimer, procalcitonin, CRP, Ferritin and LDH. However, we did not find any inter-group differences between LVO and non-LVO groups for any of these markers except ferritin. The LVO group had significantly higher levels of ferritin than non-LVO patients. Ferritin is known to be a marker for severity of COVID-19, although its significance with regards to neurovascular events has not been clearly delineated (12).

In terms of etiological distributions, we encountered the gamut of small vessel occlusions, cardiogenic emboli, large artery atherosclerosis as well as stroke of undetermined etiology. The most common were the cryptogenic category (6 patients) followed by small vessel occlusions and unclassified-undetermined etiologic category (3 patients). Our results were thus similar to Yaghi et al. with undetermined being the most common etiology in our analysis (50%) of which cryptogenic predominated (20). However, we often found it difficult to ascribe causative mechanisms in our series; at times due to incomplete testing and other times due to multifactorial pathogenesis of stroke in these cases. This is illustrated particularly by cases which were difficult to ascribe to a single etiology or developed strokes in spite of aggressive medical therapy (Patients #8, #10, and # 14). All of them exhibited elevations of inflammatory markers. Thus, it is possible that COVID-19 related thromboembolism may follow multiple separate pathogenetic pathways. In patients with pre-existing vascular risk factors, it may predispose to an ischemic event earlier than if purely driven by those underlying conditions. This could explain why some younger patients in the 20–55 age range presented with stroke. In these situations, it may follow the paradigm seen in other hypercoagulable conditions where two or more factors act synergistically to increase risk of stroke (14). It is also possible that in some cases this hypercoagulable condition by itself is enough to cause an embolic stroke. This may be correlated with elevated lab markers like D-Dimer or NLR or presence of antiphospholipid antibodies (10, 30). A third pathway may be related to its effect on the heart when it causes cardiomyopathy or myocarditis which in turn may predispose to cardioembolism (18, 36). This mechanism may be mediated by ACE2 targeting which could affect the vascular endothelium or the heart directly. Finally, since the incidence of VTE is higher in these cases, the possibility of paradoxical emboli cannot be ignored (14). To our knowledge, this is the first study that attempts to differentiate between the LVO and non-LVO phenotypes of ischemic strokes associated with COVID-19 and discuss their presentation, diagnostic data, etiology and outcome within that context.

### Limitations

Our study has several limitations. This is an inherently small analysis of neurovascular cases presenting to two hospitals. As the number of cases in our hospitals often overwhelmed the capacity for testing or intensive care during the initial surge, many were not tested. Thus, we cannot determine the true incidence rate of neurovascular events in COVID patients. Due to the stress on the resources created by COVID-19 some of the patients did not receive advanced imaging such as MRI to confirm whether unknown infarcts may have occurred. Additionally, many cases had other risk factors and the embolic events may have been related to the underlying risk factor rather than the viral infection, or simply exacerbated by it. Finally, the type of strokes caused by the disease fit into several etiologic pathways rendering it difficult to delineate an appropriate treatment or prevention strategy.

## Conclusion

Stroke in the setting of COVID-19 has an unusual presentation including atypical demographics and delayed time windows. COVID-19 related ischemic events can present as small vessel occlusions, multi-territorial embolic infarcts or large vessel occlusions. The most common etiology is cryptogenic followed by undetermined secondary to multiple mechanisms. Ischemic stroke can be a presenting symptom of COVID-19 and may not always be associated with severe disease markers including in the young, minorities and frontline workers.

## Data Availability Statement

All datasets presented in this study are included in the article/Supplementary Material.

## Ethics Statement

Study regarding human participants were reviewed and approved by the IRB committees at Brookdale Hospital University Medical Center & Jamaica Medical Center, respectively. Written informed consent from the patients/next of kin was not required to participate in this study in accordance with the national legislation and the institutional requirements.

## Author Contributions

AT was responsible for concept, data collection, review as well as writing the manuscript. AD and KA were responsible for concept, review as well as editing the final manuscript. SK, KH, MS, AL, TN, TZ, and DK were responsible for clinical care and review of the manuscript. JFr and SY were responsible for clinical care, editing, restructuring, and review of the manuscript. DT-P, JL, and JFa were responsible for the cases and review of the manuscript. VV, SA, and AK were responsible for data collection and review of the manuscript. KB was responsible for data collection, review as well as writing the manuscript. All authors contributed to the article and approved the submitted version.

## Conflict of Interest

The authors declare that the research was conducted in the absence of any commercial or financial relationships that could be construed as a potential conflict of interest.
