# Supplementary material for: Etiologic Subtypes of Ischemic Stroke in SARS-CoV-2 Patients in a Cohort of New York City Hospitals
Source: Front Neurol. 2020 Sep 17;11:1004. doi: 10.3389/fneur.2020.01004 (PMC7527497; doi:10.3389/fneur.2020.01004)
Supplement: Supplementary file 1 [file Data_Sheet_1.PDF]

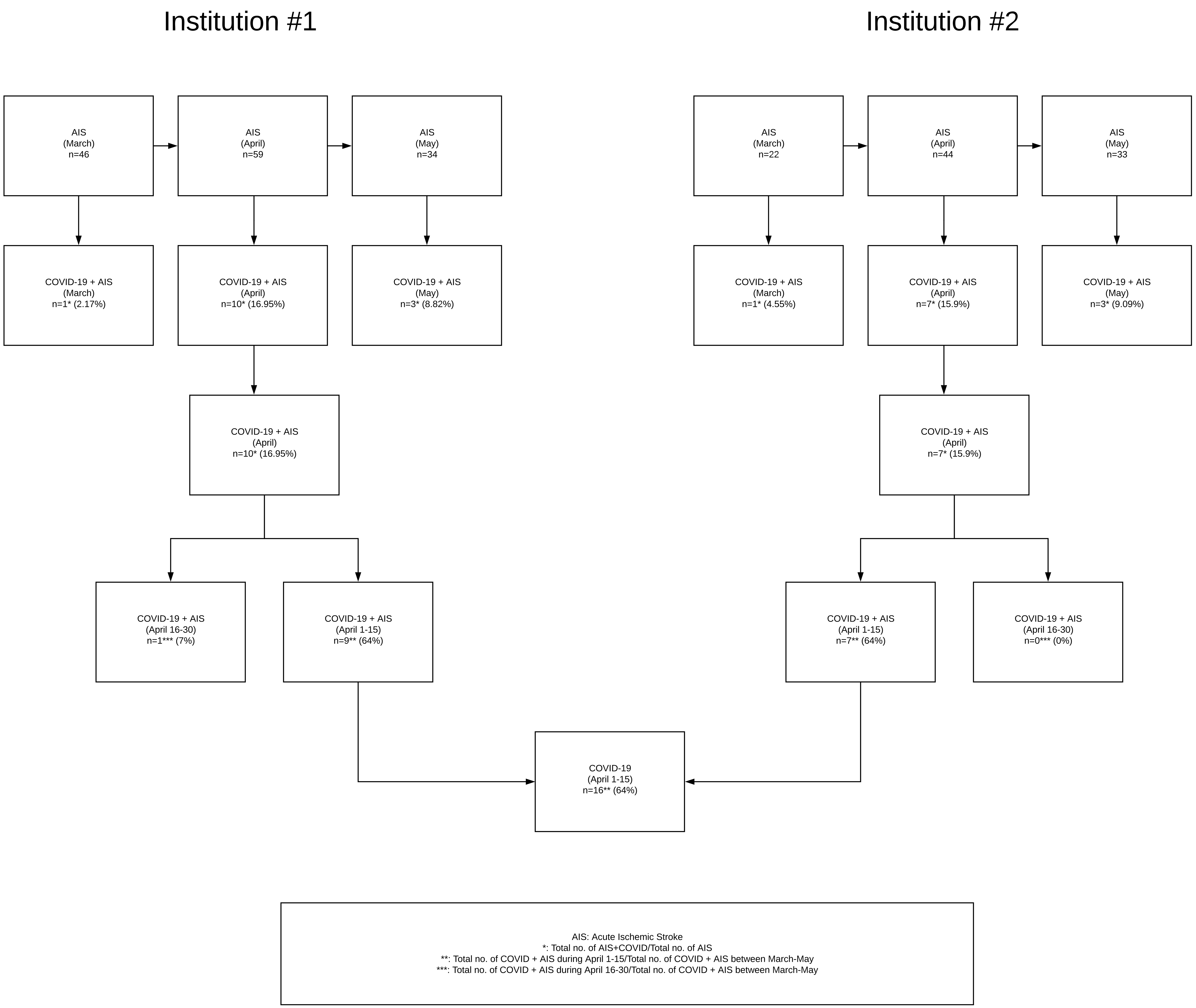
Institution #1
Institution #2
AIS
(March)
n=46
AIS
(April)
n=59
AIS
(May)
n=34
COVID-19 + AIS
(March)
n=1* (2.17%)
COVID-19 + AIS
(April)
n=10* (16.95%)
COVID-19 + AIS
(May)
n=3* (8.82%)
COVID-19 + AIS
(April)
n=10* (16.95%)
COVID-19 + AIS
(April 16-30)
n=1*** (7%)
COVID-19 + AIS
(April 1-15)
n=9** (64%)
AIS
(March)
n=22
AIS
(April)
n=44
AIS
(May)
n=33
COVID-19 + AIS
(March)
n=1* (4.55%)
COVID-19 + AIS
(April)
n=7* (15.9%)
COVID-19 + AIS
(May)
n=3* (9.09%)
COVID-19 + AIS
(April)
n=7* (15.9%)
COVID-19 + AIS
(April 1-15)
n=7** (64%)
COVID-19 + AIS
(April 16-30)
n=0*** (0%)
COVID-19
(April 1-15)
n=16** (64%)
AIS: Acute Ischemic Stroke
*: Total no. of AIS+COVID/Total no. of AIS
**: Total no. of COVID + AIS during April 1-15/Total no. of COVID + AIS between March-May
***: Total no. of COVID + AIS during April 16-30/Total no. of COVID + AIS between March-May
